# Bridging the silos in HIV and Hepatitis C prevention: a cross-provincial qualitative study

**DOI:** 10.1007/s00038-016-0914-9

**Published:** 2016-10-25

**Authors:** Anik Dube, Greg Harris, Jacqueline Gahagan, Shelley Doucet

**Affiliations:** 1 School of Nursing, Universite de Moncton, Moncton, NB Canada; 2 Faculty of Education, Memorial University of Newfoundland, St. John’s, NL Canada; 3 School of Health and Human Performance, Dalhousie University, Halifax, NS Canada; 4 Nursing and Health Sciences, University of New Brunswick, Saint John, NB Canada

**Keywords:** Intersectoral collaboration, Gender, HIV and HCV prevention

## Abstract

**Objectives:**

The Our Youth Our Response (OYOR) study explored the scope and accessibility of existing youth-oriented human immunodeficiency virus (HIV) and Hepatitis C (HCV) prevention in Atlantic Canada.

**Methods:**

A cross-provincial, qualitative population health and gender-based analytic approach was used in this study. Four hundred and twenty-five documents were part of the initial scoping review, while 47 in-depth interviews across youth-relevant sectors were undertaken to explore the perceptions related to current approaches to youth-oriented HIV/HCV prevention policies and programs. The study also conducted focus group discussions with 21 key informants aimed at identifying strategies to address the challenges identified from the interview data.

**Results:**

Five overarching themes emerged from our triangulated data in relation to the present state of youth-related HIV/HCV prevention. These included: inter-organizational and intersectoral collaboration; youth engagement; access to testing; harm reduction; and education.

**Conclusions:**

Our findings will assist in informing the next generation for HIV/HCV prevention aimed at youth. Specifically, the results indicate that future prevention initiatives should support the use of intersectoral collaboration, gender-based approaches, and HIV/HCV testing innovation to help de-stigmatize prevention efforts.

## Introduction

Despite recent advances in treatments for HIV and HCV, prevention of these blood-borne infections remains a significant public health issue in Canada and globally (Tucker et al. 2013). Further, these preventable blood-borne viruses create significant social and financial burdens, not only for the infected individuals, but also for prevention services. This is particularly challenging for populations of at-risk youth (e.g., street involved, injection drug using, MSM, those under the age of 30). In 2011, the Public Health Agency of Canada (PHAC) estimated that 71,300 Canadians were living with HIV, an increase from 64,000 in 2008 (CATIE, 2015; PHAC 2014a). Of these, female youth aged 15–29 years account for 26% of all reported HIV cases in Canada, while male youth account for 24% of reported cases (PHAC 2014a). As of 2012, the national HCV infection rate was estimated at 245,987, with 332,414 individuals testing positive for HCV antibody (PHAC 2014b). Male youth aged 15–24 are diagnosed at a rate of 32.1 per 100,000 individuals, while female youth are diagnosed at 43.3 per 100,000 individuals (PHAC 2014b). As indicated by these data, young people in Canada remain a population group at risk of contracting HIV and/or HCV.

It is important to note that a complex array of various determinants of health at both the individual and broader structural level create challenges in the prevention of HIV/HCV. As suggested by Raphael (2008), the social determinants of health such as poverty, social exclusion, housing, food insecurity, among others, impact on youth’s likelihood of contracting HIV/HCV. Specifically, youth face challenges in accessing youth-friendly information and services on negotiating safer sexual intercourse and safe injection drug use (IDU). In addition, youth experience gender-based stereotype in prevention programs, which has been identified as significant contributors to social marginalization and stigmatization among those seeking access to HIV/HCV prevention services (Gahagan et al. 2011; Shoveller et al. 2009). Given these challenges, our study explored the current state of primary and secondary HIV/HCV prevention initiatives targeting youth 15–24 years of age in the Atlantic region of Canada, using population health, determinants of health, and gender-based conceptual perspectives (McLeroy et al. 1998; PHAC 2010). Atlantic Canada was targeted for this research and is comprised of four provinces: New Brunswick, Newfoundland and Labrador, Nova Scotia, and Prince Edward Island. It is noteworthy that, relative to the rest of Canada, people living in the Atlantic region experience significantly higher burdens of chronic disease, higher levels of poverty, homelessness and food insecurity (Knezevic et al. 2014; Murray et al. 2013; PHAC 2009). They also have geographic barriers such as limited access to specialized healthcare, limited resources, and a significant proportion of rural regions (Murray et al. 2013). Youth in Atlantic Canada and elsewhere in the world are particularly vulnerable to these conditions, as many face risk of HIV/HCV infections due to social stigma related to youth sexuality, barriers to access services, limited education pertaining to risks of exposure, and lack of youth-oriented prevention initiatives (Gahagan et al. 2011). It is, therefore, imperative that the impact of these social, economic, and political contexts be examined and addressed, particularly in relation to the prevention of HIV/HCV infections to inform provincial, national, and international health related policies and programs targeting youth and at-risk populations.

The complexities of preventing blood-borne infections among youth are well known. In fact, many international health organizations have recommended the use of a multi-level, collaborative approach to patient-centered care for this population to address these complexities (Chircop et al. 2014; Frankish et al. 2007; World Health Organization 2014). This approach is consistent with the World Health Organization’s (WHO) vision for healthcare, which advocates for intersectoral action for health. WHO defines the intersectoral concept as a “relationship between part or parts of the health sector with parts of another sector which has been formed to take action on an issue to achieve health outcomes… that is more effective, efficient or sustainable than could be achieved by the health sector acting alone” (WHO 1997, p.3). Intersectoral collaborations are a dynamic process of interaction that occurs during information exchange, policy development, and program-planning activities related to prevention (Fuster et al. 2011; Harris et al. 2012). These collaborations also allow service providers to share their expertise and resources to identify and address the key determinants influencing the health status of youth. This approach has not been adopted by many Canadian provinces. Further, the health system is functioning in a fractured way according to O’Reilly (2014). This is reflected in the Romanow report (2002), which suggests that minimal efforts are being made to solidify the complex intersections between all micro- to macro-level factors influencing, for example, access to healthcare (O’Reilly 2014). While functioning in this fractured way, different sectors may be unaware of their duplicated efforts and the potential impact this may have on healthcare costs (Dick and Ferguson 2015).

Within intersectoral collaboration is the urgent need for greater conceptual and practical clarity on key concepts such as sex and gender. These concepts are often seen as pivotal to the discourse on disease prevention, from epidemiological surveillance data reporting to research opportunities (Gahagan 2016; Gahagan et al. 2011; Shoveller et al. 2009). Given that sex and gender, however, carry distinctive, yet often conflated, characteristics, these are important factors to consider in HIV/HCV prevention in that it relates to the physiological differences between males and females, including susceptibility and disease progression. Unlike sex, gender is a relational concept that intersects with other social determinants, such as the influence of age, literacy levels, access to and uptake of preventative services for HIV/HCV. Gender as a key determinant can, for example, influence patient care across sectors, which includes prevention initiatives, treatment delivery, and access (Shoveller et al. 2009). Gender roles, including expectations about how young men and women should socially behave and relate their sexuality in particular context, also become influential factors in how, and indeed if, youth will access services (Gahagan et al. 2011; Johnson and Repta 2012). When interacting with others (e.g., health service providers), young men and women often struggle internally with their self-perceived masculinity or femininity (Johnson and Repta 2012). Gender-based differences can create vulnerabilities and inequities, such as social marginalization and stigmatization, while defining or re-defining roles and responsibilities ascribed by an underlying understanding of what is acceptable in a given society (Gahagan et al. 2011). Therefore, neglecting to acknowledge the impact of a key determinant, such as gender, may mean missing critical barriers to access that influence the risk of contracting an infectious disease. The current paper focuses on the study findings related to the five overarching themes, with a particular focus on intersectoral collaborations and gender-based approaches as a means to enhance HIV/HCV prevention efforts aimed at at-risk youth globally.

## Methods

The current exploratory study focused on the scope and accessibility of existing youth-oriented HIV/HCV prevention initiatives based on population health, determinants of health and gender-based perspectives in Atlantic Canada. The study also identified gaps and weaknesses in the present prevention policies and programs. The research was conducted in collaboration with community-based organizations, an interprofessional research team and a youth advisory committee made up of representatives from each province. These groups served an integral role in providing insight into youth-focused HIV/HCV programs. The groups also helped to disseminate recruitment materials as well as research findings through their respective networks. This study was divided into three phases. During the first phase, the research team completed a scoping review of policy documents on youth-oriented prevention of HIV/HCV and published the findings (Hare et al. 2015). To triangulate the data, the findings from the initial scoping review were used to inform phase two and three, which included the development of the interview guide and the subsequent focus group guide.

In phase two, 47 key informants from all sectors were interviewed using semi-structured, in-depth interviews to explore their perceptions of themes related to primary and secondary prevention of HIV, gender, sex, diversity, and equity. Participants were recruited using a purposive sampling strategy by sectors and background. Potential participants contacted the research assistants to arrange one-on-one interviews. Individuals were invited to participate in an interview if they were a youth, government, healthcare policy decision maker, health service provider, or educator for a youth-focused HIV/HCV service organization (Table 1). The inclusion criteria required participants to have knowledge of HIV and/or HCV prevention policy or programming issues for youth, to have lived and worked in Atlantic Canada, and being sixteen years of age or older. Once the participant read, accepted and signed the consent, interviews were audio-recorded and conducted by phone or in person, and then transcribed verbatim using Nvivo9. Coding was done iteratively in collaboration with other provincial members using a pre-developed codebook. The team coded the same transcripts and then compared initial findings to help ensure inter-coder reliability. Braun and Clark’s (2006) approach to thematic analysis was used to explore how gender as a social determinant of health, as well as issues of equity pertaining to gender identity, were reflected in HIV/HCV prevention. The analysis also helped identify promising practices in each province and gaps in youth-related HIV/HCV prevention across sectors. This phase allowed the team to reflect critically on boundaries surrounding HIV/HCV initiatives and enabled them to identify innovative approaches.

**Table 1:** Summary of the number of interviewed key informants per province and per sector

Sectors	NB	NL	NS	PEI	Total	%
Education	2	5	4	1	12	26
Justice	1	0	0	1	2	4
Health	8	5	4	2	19	40
Community	4	4	3	0	11	24
Unreported	0	0	3	0	3	6
Total	15	14	14	4	47	–

The final phase of the study included four focus group discussions with 21 key informants from community-based organizations, government, health institutions, and youth sectors. The purpose of the focus group discussions was to explore potential solutions or strategies to address the barriers as indicated in the in-depth interview data and solicit feedback on how best to advance HIV/HCV prevention policies and programs among youth. A purposive sampling method was also used for recruitment with a balanced mix of representatives from community-based organizations, government health departments, service providers, and clients. Following the consent procedure, focus group discussions were audio-recorded, transcribed, and managed with Nvivo9. The focus group codebook was derived from, and expanded upon, the in-depth interview data to reflect emergent themes identified during phase two. Lessons learned, and best practices were also factored into the focus group analysis, to identify gaps and barriers, as well as identify practical opportunities to collaborate and to look for ways to maximize existing resources. Following the analysis of the in-depth interviews and focus groups, key themes emerged in relation to the prevention landscape.

## Results

The five emergent themes in relation to youth-oriented HIV/HCV prevention were identified: (1) inter-organizational and intersectoral collaboration; (2) youth engagement; (3) access to testing; (4) harm reduction; and (5) education. Quotes have been categorized by provinces and by participants (NB: New Brunswick participant; NL: Newfoundland and Labrador participant; NS: Nova Scotia participant; and PEI: Prince Edward Island participant). As indicated in the following, participants often quoted intersectoral collaboration as the most beneficial way to enhance HIV/HCV prevention. A significant, overarching finding was the absence of gender, as a key determinant of health, from most youth-oriented HIV/HCV prevention policies and programs.

### Inter-organizational and intersectoral collaboration

During the study, participants were asked to reflect on the level of collaboration used in the creation or implementation of youth-focused HIV/HCV prevention initiatives. This question was aimed at elucidating the degree of collaboration between the various sectors. Most participants indicated that collaboration between, and across sectors were critical to the delivery and efficiency of gender-based policies and programs, in keeping with the requirements of funding bodies and the government of Canada (Government of Canada 2016). Some organizations had ongoing efforts about collaboration and gender-specific strategies, and many indicated a desire for initiating these efforts. Most stated that strategic partnerships across sectors and at-risk populations, such as homeless youth, lesbian/gay/bisexual/transgender/queer (LGBTQ) youth, and incarcerated youth, would improve collective efforts in meeting gender-oriented and youth-related needs. According to one key informant working in public health prevention programs, there is a present increase in the number of young females becoming infected with HCV through IDU. As indicated below, informing key partners of this important gender variance and collaborating with at-risk populations would help better target prevention initiatives and enhance the prevention of HCV across sectors, however, time is a barrier to sometimes initiating inter-organizational and intersectoral collaborative efforts.

Well, if you look at hepatitis C, it used to be that a lot more males were testing positive for HCV… In our latest infectious disease report, we are now looking at almost a 50% ratio of females testing positive for this disease. It never used to be this way. This is telling us something. We now have as many females doing intravenous drugs in the province than we do men… The culture of doing intravenous drugs has also changed. Maybe females were more scared of doing it [IDU] and now they are getting more relaxed about it. They are getting much younger and health providers need to know how to react… However, I have little time to call a meeting with my colleagues from different organizations to discuss this [gender] variance happening with hepatitis C. [NB10]

### Youth engagement

Increasing the level of youth engagement in the development and implementation of HIV/HCV prevention initiatives can help increase program utilization, leading to services that better reflect the needs of diverse populations of youth. In addition, participants indicated that collaboratively engaging young people in policy and program development could serve as a means to help youth to further build skills and make connections to enhance access and advocate for needed HIV/HCV prevention services.

We always let the kids know that this is their group; it is not our group… And so you just let them run their group their way. We just come in and present a topic each night. But then we just [leave] them for the second hour… when we come to clean up, all the condoms have been blown up, and are hanging from the chandelier. They just have fun with it… Kids will be kids, and you have to allow them that opportunity to really be themselves because they express more with each other than they will with adults. [NS Focus Group 1]

### Testing

According to our participants, access to timely, gender-appropriate, and youth-related HIV/HCV testing services is a significant barrier to early diagnosis and timely referral to treatment. Access to these testing services is an important public health issue for all provinces. Participants indicated that where testing services exist, they are often inaccessible, creating unnecessary levels of barriers among youth seeking testing. Participants also discussed that many youth were hesitant to seek out testing at non-dedicated service locations such as walk-in clinics due to social stigma.

…I think we would need to make sure that the testing programs were in place, and that they were easily accessible, that youth can drop in and that services are friendly and sensitive to different gender related issues, such as confusion related to identity. The services and stuff should actually be designed by youth. I think sometimes that failure of people to access a program means that we did not need the program. When in fact, we just did not design the program in consultation with the people [youth] we were hoping to serve and the needs we were hoping to meet. [NB1]

### Harm reduction

Harm reduction perceptions and institutional policies, along with public misinformation about these programs, are seen by many participants as structural or system-level barriers in addressing the needs of at-risk youth. Participants indicated that access to non-judgmental services without gender-based stereotypes and gender-based inequities could help address broader, structural determinants of health perspective such as socio-economic, housing, and education in an effort to remain inclusive and responsive to the complex needs of diverse populations.

A lot of people misunderstand the term harm reduction. If you do not understand it and then you get bent out of shape because a particular group is actually providing these services, you can get into a lot of trouble when it comes to your funder, your neighborhoods or wherever your office is. Because if your neighbors find out that you are actually giving needles out or giving out free condoms out, they basically say, oh, we are just encouraging that kind of activity among a high risk group of youth [homeless and LGBTQ youth]… [NS Focus Group 1]

…I will inform them [youth who use injection drugs] where, if they have to have new needles, where they can go to get them. I dont know that it is publicly advertised where these needle exchange sites are. This might be a bit of a barrier. [PEI2]

### Education

Increasing awareness about HIV/HCV cannot only help to inform the people about the importance of prevention, but it can also help correct misconceptions related to HIV/HCV testing and treatment options. According to our participants, there are more prevention initiatives related to HCV compared with HIV. In addition, the lack of a guiding policy for present programs has led to discordance across sectors.

We need to focus more on all youth and right now, we are not [sexual health education]… We also feel that there is a lack in teaching parents about how to talk to their children about sexual health. Public health is developing a program that hopefully will be able to implement and give to parents on how to talk to their kids… I’ve delivered such a program to high school and it was very successful. The program was organized in collaboration with the youth who identified as either being straight, gay, lesbian, or bisexual… the parents have to be involved also in learning how to feel more comfortable, because they should be the main educators of their children when it comes to sexual health and sexual identities. [NB13]

## Discussion

In this study, we investigated youth-based HIV/HCV prevention through the multi-level population, determinants of health and gender-based lenses across the four Atlantic provinces of Canada. This approach allowed the research team to analyze the current state of HIV/HCV prevention programs and policies with an eye to soliciting diverse perspectives across sectors. An additional goal was to provide an HIV/HCV prevention-focused capacity-building opportunity. This effort would help ensure that policy-to-programming approaches adequately meet the HIV/HCV prevention needs of at-risk youth.

### Recommendations

Our results highlight the significance of intersectoral collaboration and gender-based approaches as critical components to building a strong platform for youth-related HIV/HCV prevention. Enhancing intersectoral collaboration allows decision makers and service providers to connect with a range of youth populations and provide more comprehensive prevention initiatives, while at the same time acknowledging the complex and intersecting determinants of HIV/HCV prevention (Harris et al. 2012; Lawless et al. 2012). Most critically, participants urged the integration of intersectoral collaboration in all disease prevention mandates. This collaborative approach can enable service providers working in different sectors to meet, share, and innovate on a more regular basis. According to our study participants, a need for greater strategic partnerships with governments and collective sectoral initiatives are imperative. This finding is crucial for Canadian policy and programming development but has significant implications globally.

Internationally, advocates for intersectoral collaboration recommend opening structures to view sectors and organizations a system (Adams et al. 2014; PHAC 2008) that can be molded and changed to meet the shifting prevention needs of a given community or population. Many different regions in Canada and other places in the world apply innovative approaches to prevention among youth. For example, the Joint United Nations Programme on HIV/AIDS states that collaborative HIV strategies must be informed by evidence and complemented with concurrent social, clinical, and structural prevention efforts (Adams et al. 2014). To enhance the significance of integrated prevention approaches, a multi-level consultation facilitated a collective conversation on some innovative gender-focused strategies across HIV research, services, and public/private partnerships. Several recommendations emerged from this international consultation including enabling intersectoral collaboration to evolve and enhance contextual prevention initiatives (Adams et al. 2014). Harris et al. (2012) also encourage collaboration within and beyond the health sector by engaging others, as there is a growing need for a structured space for intersectoral engagement in healthcare.

Throughout this study, participants articulated the importance of having the perspectives of diverse populations of youth to enhance access to testing, harm reduction services, and gender-based education. Our findings indicate that the input and involvement of youth can improve the reach of programs within the context of gender-based perspectives in HIV/HCV prevention. Although considered essential by many, actual sustained youth engagement remains relatively limited. Reed and Miller (2014) agree that youth are often excluded from various health promotion and disease prevention activities and planning initiatives. They suggest that negative attitudes among adults are a significant barrier to youth inclusion, as adults often view young people as lacking credible knowledge to inform initiatives. A solution to this tension would be to have a clearly articulated prevention mandate for youth engagement across sectors, ensuring accessible, innovative, and confidential testing services, along with harm reduction services for youth. Our findings indicated that increasing awareness across sectors and informing health service providers and educators, to correct misconceptions related to gender and HIV/HCV (e.g., homosexuality is the leading cause of HIV and heterosexuals in relationships do not require HIV/HCV testing) leads to increased consistency in targeted approaches to HIV/HCV prevention. Building awareness in partnership with youth and enhancing participation in initiatives across sectors are essential elements in ensuring the success of youth-focused HIV/HCV prevention (Schulman 2006).

Although many participants saw the importance of gender as a key social determinant of health, there were different perspectives on how to address gender in HIV/HCV prevention efforts aimed at youth. Importantly, the different levels of understanding among various stakeholders, combined with the lack of integration of gender-based perspectives in various policies and programs, are influential factors in the uptake of various prevention initiatives. Further, most participants did not articulate gender as a relational concept constructed with other people and places, but rather they defined gender using a gender binary for their understanding of males and females. Therefore, many existing HIV/HCV prevention policies and programs appear to be missing this critical element of gender in prevention. The concept of gender is an important social factor in the fight against HIV/HCV, as it has been associated with many of the reasons why youth choose not to access testing services in their communities or healthcare settings (Shoveller et al. 2009; WHO 2014). In addition, many international organizations, such as the United Nations, the WHO, along with the Auditor General of Canada and the House of Commons Standing Committee on the Status of Women, have strongly emphasized that policy development in disease prevention needs to reflect gender-based differences in all sectors (Canadian Institutes of Health Research 2014; WHO 2015). From a prevention policy to programmatic perspective and health advisors across different sectors could benefit from a clear understanding of gender, as it is often misconstrued in existing programs.

## Conclusion

In conclusion, participants in this study strongly urged the bridging of silos across sectors, in an effort to prevent duplication of HIV/HCV prevention policies and programs while streamlining integrated initiatives. Our analysis revealed that for many stakeholders, their sector of practice, their core mandate, and the region in which they work influenced their ability to work in collaboration with others. In many cases, public health agencies and organizations, such as ASOs, were very efficient in tapping into national awareness campaigns by working in collaboration in building youth-friendly integrated HIV/HCV prevention messaging. This approach was seen as critical to unpacking barriers, such as gender-based stereotypes, resource constraints, and limited access, while enhancing the understanding of youth-focused approaches, such as safe spaces for prevention, e-health campaigns, and non-judgmental educational and innovative testing initiatives. Although used strategically in certain sectors and regions, not all programs mandated intersectoral collaboration and gender-based approaches, which hindered efforts to strengthen current HIV/HCV prevention for youth. Therefore, to facilitate a successful scaling up of collective approaches, stakeholders and target populations will need to forge strategic partnerships to better meet the prevention needs of youth. Moving forward, strategic efforts should be aimed at normalizing and facilitating intersectoral collaboration, including gender-based analyses of innovative HIV/HCV testing approaches to help de-stigmatize prevention efforts aimed at at-risk youth.
